# Psychiatry, colonialism and Indian civilization: A historical appraisal

**DOI:** 10.4103/0019-5545.31600

**Published:** 2006

**Authors:** Shridhar Sharma

**Affiliations:** * Professor, Emeritus National Academy of Medical Sciences and Institute of Human Behaviour and Allied Sciences, Delhi e-mail: sharma.shridhar@gmail.com

Dr Basu's paper on ‘Historicizing Indian psychiatry’^1^ is a wonderful read, written in a lucid and powerful prose. While some authors may write better prose than many trained historians, they do not necessarily possess the rigour and insight which a trained historian brings to the subject. Scientific evaluation of social–medical–historical facts is a complex process, and must be ideally based on rational, objective and transparent criteria to help generate valid conclusions. It is also true that assessment of historical facts is often guided by one's ideology, experience and knowledge in the field. Apparently faulty assessment and subjective bias is not uncommon in the field of history while arriving at a conclusion.

It is apparent that Dr Basu's intent is more in showing the impact of colonization than contribution of other factors in the development of psychiatry in India. Secondly, the paper is based heavily on Michael Foucault's work and his views on development of psychiatric thought, which is biased by his anti-psychiatry ideology. Further, Foucault's good work was focused mainly on the 'interpretation of madness in the context of European civilization' and not of Indian civilization.

Thirdly, the paper is also greatly influenced by the belief that history writing has been decided by the West, which is conventionally considered as a repository of value and truth with an absolute right to define what is truth and what is exciting, including the culture and social issues of a non-western country. Though strange, this notion is a fact. Fourthly, those who work in the field of psychology are familiar with the faulty perception which is influenced by personal biases. The uniqueness of psychiatry as a medical specialty lies in the fact that there is a biological and psychosocial dichotomy. It is necessary for historians to appreciate this dichotomy to understand the phenomena of mental illness. There is something unique about the field of psychiatry, as psychiatry is primarily a medical specialty; yet, it has close proximity with the social sciences and cultural issues. This causes some discomfort to those who are not sensitive to these issues. To illustrate, a typical western writer's attitude is well-reflected in the views of W.S. Shaw (1932)^2^ who stated that ‘in spite of over-crowding of mental hospitals the absence of a definitely expressed public opinion continues to delay improvement under present condition of government in India and the noisy section of population led by Mahatma Gandhi who prefers the Ayurvedic and other indigenous systems to our modern methods of treatment’. Such conclusions are similar to the views that the first freedom movement of 1857 in India was perceived as ‘Mutiny’ by western writers.

Keeping the above views, we will examine the basic concept of mind and mental health in the context of colonization of India. It is true that during the period of colonization some elements of science were brought to psychiatry, but philosophy, culture and religion were already deep-rooted in the Indian tradition. James Mills,^3^ a historian, states that ‘a concern with mental health has long been a part of Indian cultures which evolved a variety of ways of attempting to understand and negotiate psychological disorder’. History is a kind of screen in which the past lightens the present and the present brightens the future. The ancient Indian thought emphasized the theory of unity of body and soul and also explained how to deal with the health and mental health problems in psychosomatic way. These concepts are part of Indian history and not borrowed from colonial rulers.

To depend totally on the western viewpoint that all cultures (except the West) construct history as a myth rather than on empirical facts will not lead us to the meaning of history, because meaning itself cannot derive solely from the western viewpoint. Eminent Indian philosopher S. Radhakrishnan^4^ believed in the idea that ‘Indian thought is a chapter of the history of human mind, full of vital meaning for us’. He further explained that ‘the ideas of great thinkers are never obsolete. The most ancient fancies sometimes startle us by their strikingly modern character for insight does not depend on modernity.’ Philosophy in India is essentially spiritual. The spiritual motive dominates life in India. The ultimate truths are truths of spirit and actual life has to be defined in their light.

Indian psychology realized the value of concentration and looked upon it as the means for the perception of the truth. It recognized close connection of the mind and the body. The yoga system of philosophy dealt in depth with both the theory and practice as applicable to mental health. Ancient Indian literature is also replete with the theory and practice of various psychotherapeutic techniques. However, a major part of ancient medical history still remains shrouded in legends. To sift historical truth from these legends certainly needs an imaginative penetration into the subject. Today, many Indian classics are either unavailable or those available are written in Sanskrit, many of which have not been translated into other Indian and European languages. Some of the psychotherapeutic techniques described in ancient literature have clearly distinct and well-defined approaches for use in various psychic disorders. A systemic comparison between these ancient methods and current psychotherapeutic techniques would be enriching to both and further enhance their applicability and acceptability. Yoga therapy and transcendental meditation are now universally well-known techniques that form a part of many traditional psychotherapeutic practices still practised in India.

Two well-known Ayurvedic treatises are: the *Charaka samhita* by Charaka, a physician and the *Sushruta samhita* by Sushruta, a surgeon. Of the four Vedas, which are supposed to be the oldest books known to the library of mankind, the Atharvaveda especially contains descriptions about the various medical problems and the concept of health and mental ill-health. The most fascinating contribution of Ayurveda is about understanding the phenomenology of diseases, where a systematic attempt was made to classify the diseases into 8 broad disciplines. It was thought that diseases result due to an imbalance of one or more of the 'humours'. Each disease was supposed to be influenced by a specific type of humour. It deserves mention that the Ayurvedic writings antedated the humoural theory of Hippocrates. Another interesting contribution of the Ayurveda was its knowledge regarding the relationship of diet and diseases and the association of a disease with a specific physical constitution.

The approach to training in Ayurveda is holistic and integrated. The system explains the state of health and disease, which is to be based on the interplay of the constituent elements of the body, the general and alimentary regimen, and the influences of time and the season.

In the field of materia medica and pharmacy, the properties of drugs and foods were investigated. Diagnosis was to be made by the five senses and supplemented by interrogation. Diagnosis was based on cause *(nidana),* premonitory indications *(purva-rupa),* symptoms *(rupa),* therapeutic tests *(upashaya)* and natural history of the development of the disease *(samprapti).* According to Sushruta, the physician *(chikitshak),* the drug *(dravya),* the attendants or the nursing personnel *(upasthata),* and the patient *(rogi)* are the four pillars on which rests the success of the therapy. The use of massages with herbal medicine and oils was also prescribed in the treatment of some diseases.

The highest patronage to the science of Ayurveda was given by the Buddhist kings (400–200 BC). With the spread of Buddhism, Ayurveda also spread to other Southeast Asian countries and was adapted to the local needs and traditions.

## COLONIALISM, CIVILIZATION AND MENTAL HEALTH; IS THERE ANY RELATIONSHIP!

There is some basic inherent contradiction in colonial administration. Most historians, who depend mainly on western writers, forget that the basic purpose of any colonial administration is to exploit the local resources and to improve the economic status of the imperialist power. In no country was development a priority of the imperialist power. Their basic aim was pursuit of power and wealth for their own enrichment.^5^,^6^ A civilization is a cultural entity; it evolves continuously and is most enduring of human associations. Their ‘unique and particular essence’ is their long historical continuity. Civilization is in fact the longest story of all. Empires rise and fall, governments come and go, civilizations remain and survive, in spite of political, social, economic, or even ideological upheavals.^5^ Civilization is cultural and not composed of political entities. Even during colonization, the Indian civilization survived, which has existed on the subcontinent since at least 1500 BC. In Europe, by AD 1500, during renaissance of European culture, and with social pluralism, expanding commerce and technological advancement provided the basis for enlarging their colonial power. The expansion of the colonies was facilitated by their organization, discipline and training of troops and subsequently by superior weaponry, transport, logistics and medical services as a result of their leadership in the Industrial Revolution. The West won the world not by the superiority of ideas or values of religion but rather by its superiority in applying organized violence in most countries.^7^ According to Paul Bairoch,^8^ in 1750, China accounted for almost one-third, India for almost one-quarter and the West for less than a-fifth of the world manufacturing output. By 1860, the West's output increased by more than half (53.7%) and India's output decreased by 8.6%. It was 77.4% for the West and 1.7% for India in 1900 and remained at the level of 1.7% till 1953 after Indian Independence and was 74.6% for the West. These facts are not well-published, but are startling.

It is a matter of record that before the arrival of the British East India Company in 1700, there were no institutions to keep the insane as family supported the individual who was sick and needed help. The early establishment of mental hospitals in the Indian subcontinent reflected the needs and the demands of European patients in India during the period.^9^–^11^ Later, the development and growth of mental institutions reflected both the interest and neglect by the colonialists who ruled India for over 200 years. Mental asylums in India, as seen today, were entirely of British conception ‘except for some ancient collections of curious humanity such as that of Shahdaula's Chauhas at Gujarat and in the Punjab’.^9^,^10^ However, there is an earlier reference of some asylums in the period of Mohammad Khilji (1436–1469). There is some evidence of the presence of a mental hospital at Dhar near Mandu, Madhya Pradesh, whose physician was Maulana Fazulur Hakim.^12^ There is also evidence that modern medicine and modern hospitals were first brought to India by Portuguese during the seventeenth century in Goa, much before the British ruled India. However, the segregation of lunatics in mental asylum and their supervision were entirely of British conceptions.^11^

The early mental institutions in the Indian subcontinent were greatly influenced by the ideas and concepts prevalent in England and Europe during those days. Primarily, the mental asylums were built to protect the community and not the insane. Such asylums were constructed away from cities with high enclosures either in dilapidated buildings such as barracks left by the military men or ‘sepoyees’ of those days. It appears that lunatic asylums in India were first started to treat European soldiers employed with the East India Company.^9^,^11^

To understand the development of mental hospitals, it is relevant to know the political developments in India during that time. Probably, the latter part of the eighteenth century was the most unstable period in Indian history with the decline and fall of powerful Moghul power in Delhi, with the consequent rise of the Marathas in most of the central part and South India, the Sikhs in the north and fights for supremacy between French and English in South India. These development imparted not only political instability but also contributed to psychological and social turmoil in the Indian subcontinent.^11^ We find that the development of lunatic asylums in Calcutta, Madras and Bombay were almost parallel to these events. It is interesting to record that these three cities grew up in the beginning largely with British enterprise. The need to establish hospitals became more acute first to treat and manage Englishmen and Indian ‘sepoyees’ employed by the British East India Company. The East India Company won the first decisive war in India at Plassey in 1757. A few years later, under the leadership of Lord Clive, they won the battle of Buxar in Bihar in 1764 against Nawab Sirajjudaula. This sudden turn of fortunes went to the head of the adventurous and covetous Englishmen in Bengal. They became unbridled masters of Bengal. There was a shameless scramble for the riches; the greed and unscrupulousness of the officials of the East India Company crossed all bounds. For such undesired behaviour, Clive was censured by the British Parliament (he later committed suicide). Clive was replaced by Warren Hastings, the first Governor-General officially appointed by the British Parliament.^13^ It was during this period that development in health and mental health were first noted. There is a direct relationship of development of health and mental health during enlightened political leadership. The history and development of mental hospitals in India are well documented in the writings of Sharma,^11^ Varma,^9^ Mills^3^ and other writers.^2^,^12^,^14^

Dr Basu's paper is a provocative contribution in the field of history of psychiatry. Voltaire once remarked that ‘History is a trick the living play on the dead’.^15^ In this context, one may conclude how many of Dr Basu's provocative ironicisms are clever tricks—wit without wisdom.
